# Concurrent Psoriatic Arthritis and Ankylosing Spondylitis in a Middle-Aged Man: A Case Report With Peripheral Joint Functional and Quality of Life Assessment

**DOI:** 10.7759/cureus.79826

**Published:** 2025-02-28

**Authors:** Aswath R Deepa, Ameena A Jaleel, Anish Ancil, Samson Debbarma, Tasyoh Thampi

**Affiliations:** 1 Department of Internal Medicine, Agartala Government Medical College, Agartala, IND; 2 Department of Internal Medicine, Travancore Medicity Medical College Hospital, Kollam, IND; 3 Department of Pharmacology, Agartala Government Medical College, Agartala, IND; 4 Department of Internal Medicine, Sunrise Hospital, Kochi, IND

**Keywords:** ankylosing spondylitis, basdai, grip strength test, mri, psoriatic arthritis, quality of life, tnf inhibitors

## Abstract

The concurrent presentation of psoriatic arthritis (PsA) and ankylosing spondylitis (AS) is rare within the spondyloarthritis spectrum, presenting unique diagnostic and therapeutic challenges due to the simultaneous involvement of axial and peripheral joints. Accurate differentiation is crucial to guide appropriate treatment strategies and optimize patient outcomes.

This report describes a 38-year-old man diagnosed with concurrent PsA and AS, managed over a 12-month period with infliximab. Functional assessments included the Bath Ankylosing Spondylitis Disease Activity Index (BASDAI), Health Assessment Questionnaire (HAQ) Disability Index, Visual Analog Scale (VAS) for pain, grip strength test, and EuroQol 5-Dimension (EQ-5D) Health Utility Index. Magnetic resonance imaging (MRI) was used to monitor joint inflammation at baseline and after 12 months.

Following 12 months of infliximab therapy, the patient demonstrated significant improvements in disease activity, functional outcomes, and quality of life: BASDAI: 7.3 → 1.9; HAQ Disability Index: 2.6 → 0.9; VAS for pain: 8.5 → 2.0; grip strength (right/left): 16/14 kg → 22/20 kg; EQ-5D Health Utility Index: 0.45 → 0.8; and MRI findings: a marked reduction in sacroiliac inflammation with no new erosive changes.

This case underscores the therapeutic efficacy of infliximab in managing overlapping PsA and AS, demonstrating improvements in disease activity, functional capacity, and quality of life. The inclusion of grip strength as an objective functional outcome measure offers novel insights into treatment response evaluation in spondyloarthropathies. Further prospective studies are warranted to validate its clinical utility and establish optimized management protocols for patients with coexisting PsA and AS.

## Introduction

Psoriatic arthritis (PsA) is typically known for its peripheral joint involvement and its association with psoriasis. However, a subset of PsA patients develops axial inflammation, termed axial psoriatic arthritis (axPsA), which shares clinical and radiological features with axial spondyloarthritis (axSpA), including ankylosing spondylitis (AS) [1]. Both conditions present similarities but also have distinct differences in their genetic, clinical, radiographic, and prognostic characteristics [2]. These distinctions are critical for accurate diagnosis and treatment, as therapeutic strategies and prognoses may differ [3]. Early differentiation is essential to guide optimal treatment decisions that impact disease progression and long-term outcomes [4]. This case report describes a 38-year-old man with overlapping features of PsA and axSpA, detailing his diagnostic workup and positive response to tumor necrosis factor (TNF) inhibitor therapy [5].

## Case presentation

Patient history and initial presentation

A 38-year-old man with a 10-year history of psoriasis presented with persistent lower back pain, intermittent peripheral joint pain, and morning stiffness lasting over one hour [6]. On physical examination, there was marked tenderness over the lumbosacral spine and sacroiliac joints. A systematic tender and swollen joint count of the hands revealed the involvement of several small joints, and dactylitis was observed in two fingers [7]. Nail abnormalities, including mild pitting and onycholysis, were also noted [8]. While the clinical features of axial inflammation (e.g., bilateral sacroiliitis on magnetic resonance imaging (MRI), restricted lumbar mobility with a modified Schober's test measuring 3.1 cm (normal >5 cm), and human leukocyte antigen B27 (HLA-B27) positivity) are consistent with axSpA [9], the presence of peripheral joint findings (tenderness, swelling, dactylitis, and nail changes) supports a concomitant PsA phenotype [10]. Although psoriasis is present in approximately 10% of axSpA patients [11], the additional evidence of peripheral joint inflammation in this patient reinforces an overlapping PsA diagnosis beyond reliance on classification criteria alone [12].

Diagnostic workup and findings

The patient's inflammatory status was confirmed by elevated C-reactive protein (CRP) and erythrocyte sedimentation rate (ESR) [13]. MRI of the axial skeleton demonstrated bilateral sacroiliitis with active inflammation and subchondral edema, as well as peripheral enthesitis at the Achilles tendon insertion [14]. Although dedicated imaging of the peripheral joints (via ultrasound or MRI) was not performed due to logistical constraints, the comprehensive clinical evaluation, including detailed tender and swollen joint assessments, provided supportive evidence of peripheral synovitis [15].

Given the potential risk of reactivating latent tuberculosis (TB) with TNF inhibitor therapy, the patient underwent extensive latent TB screening, including a tuberculin skin test (TST), an interferon-gamma release assay (IGRA), and a chest X-ray; all results were negative [16]. This rigorous screening protocol is consistent with current recommendations for safely initiating biologic therapy [17].

Based on these findings, the patient fulfilled both the Assessment of SpondyloArthritis International Society (ASAS) criteria for axSpA [9] and the Classification Criteria for Psoriatic Arthritis (CASPAR) criteria for PsA [10].

Baseline functional and quality of life assessments

These results are summarized in Table 1, which provides a detailed overview of the patient's functional and quality of life assessments at baseline.

**Table 1:** Baseline scores and interpretations Functional assessments included the BASDAI, HAQ Disability Index, VAS for pain, grip strength test, and EQ-5D Health Utility Index. BASDAI: Bath Ankylosing Spondylitis Disease Activity Index; HAQ: Health Assessment Questionnaire; VAS: Visual Analog Scale; EQ-5D: EuroQol 5-Dimension; MRI: magnetic resonance imaging; axSpA: axial spondyloarthritis; PsA: psoriatic arthritis

Assessment tool	Baseline score	Interpretation
BASDAI	7.3	High disease activity
HAQ Disability Index	2.6	Severe functional disability
Grip strength (R/L hand)	16 kg/14 kg	Severe reduction in muscle strength
VAS for pain	8.5/10	Severe pain in axial and peripheral joints
EQ-5D Health Utility Index	0.45	Poor quality of life
MRI findings	Bilateral sacroiliitis, peripheral enthesitis, mild new bone formation	Confirming axSpA and PsA

Treatment and monitoring

Initiation of Biologic Therapy

Due to high disease activity and an inadequate response to non-steroidal anti-inflammatory drugs (NSAIDs) (naproxen 500 mg twice daily for six weeks), the patient was initiated on infliximab at a dose of 5 mg/kg IV [18]. The treatment followed a standard induction protocol at weeks 0, 2, and 6, with maintenance dosing every eight weeks [18].

Twelve-Month Follow-Up

At the 12-month follow-up, the patient demonstrated significant improvements across all measured outcomes (see Table 2). Improvements were evident not only in subjective measures (Bath Ankylosing Spondylitis Disease Activity Index (BASDAI), Health Assessment Questionnaire (HAQ) Disability Index, and Visual Analog Scale (VAS) for pain) [18] but also in objective functional outcomes such as grip strength and quality of life assessments [19]. MRI findings confirmed a marked reduction in sacroiliac inflammation without new erosive changes [14].

**Table 2:** Twelve-month follow-up and interpretations Functional assessments included the BASDAI, HAQ Disability Index, VAS for pain, grip strength test, and EQ-5D Health Utility Index. BASDAI: Bath Ankylosing Spondylitis Disease Activity Index; HAQ: Health Assessment Questionnaire; VAS: Visual Analog Scale; EQ-5D: EuroQol 5-Dimension; MRI: magnetic resonance imaging

Assessment tool	Baseline score	12-month score	Interpretation
BASDAI	7.3	1.9	Reduced to low disease activity
HAQ Disability Index	2.6	0.9	Marked functional improvement
Grip strength (R/L hand)	16 kg/14 kg	22 kg/20 kg	Significant strength recovery
VAS for pain	8.5/10	2.0/10	Major pain reduction
EQ-5D Health Utility Index	0.45	0.8	Improved quality of life
MRI findings	Active sacroiliitis, peripheral enthesitis	Reduced inflammation, no new erosions	Positive imaging response

**Figure 1:**
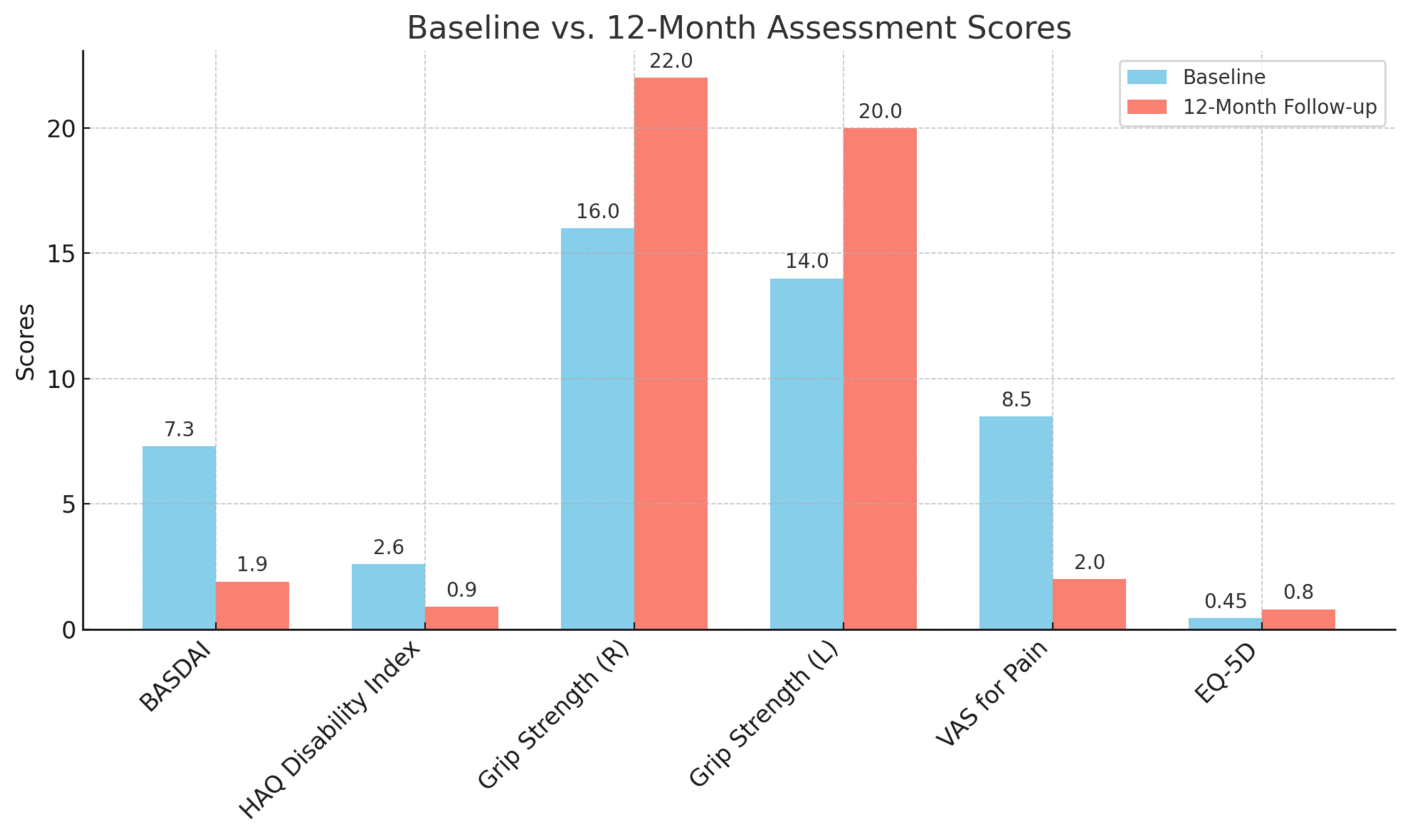
Bar chart showing baseline vs. 12-month scores Functional assessments included the BASDAI, HAQ Disability Index, VAS for pain, grip strength test, and EQ-5D Health Utility Index. BASDAI: Bath Ankylosing Spondylitis Disease Activity Index; HAQ: Health Assessment Questionnaire; VAS: Visual Analog Scale; EQ-5D: EuroQol 5-Dimension

This bar chart illustrates the comparative changes in functional and quality of life metrics between baseline and the 12-month follow-up (Figure 1).

This comparison underscores the effectiveness of infliximab in mitigating inflammation and preserving joint structure in a patient with concurrent PsA and AS.

**Figure 2:**
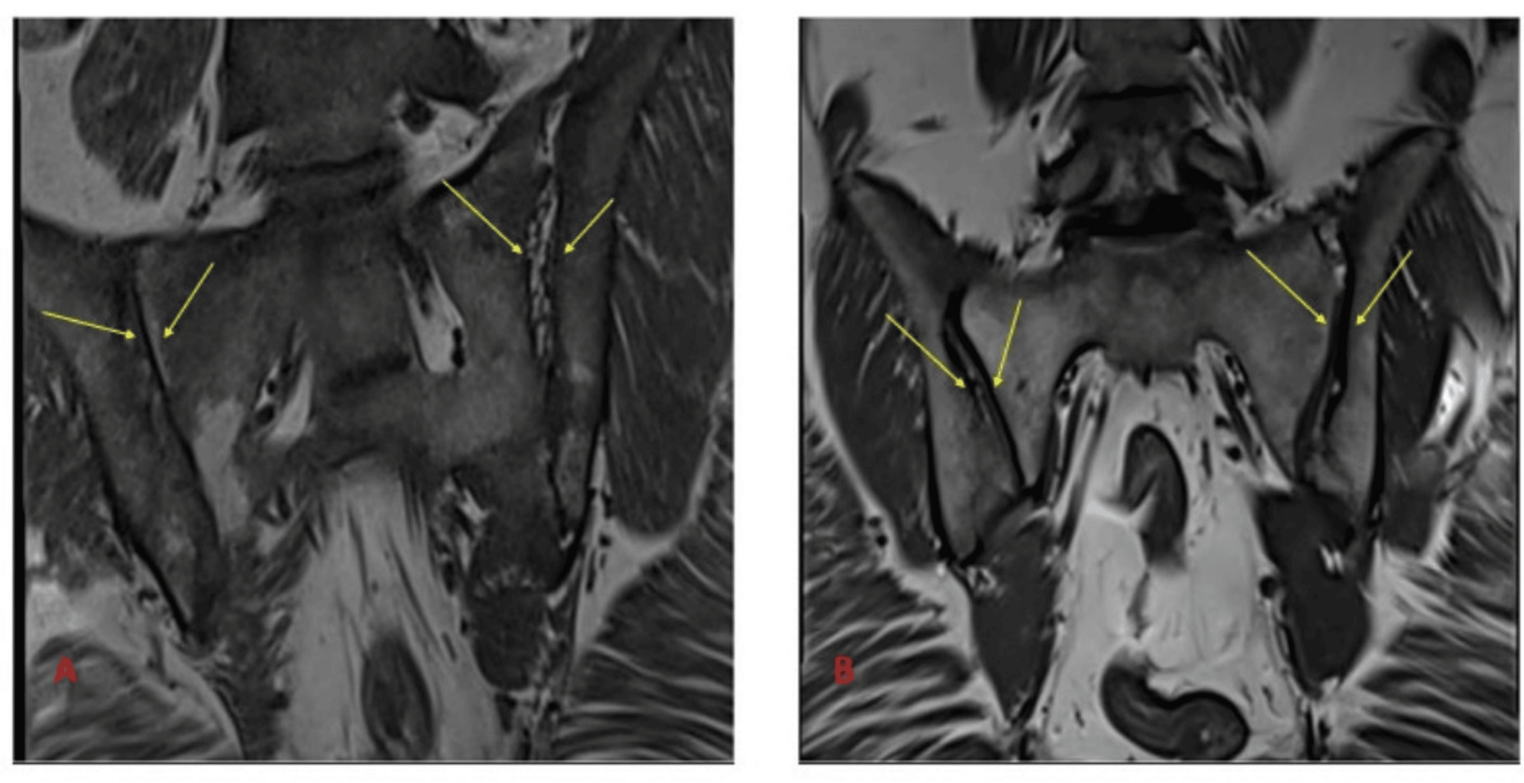
MRI T1-weighted image of sacroiliac joints before and after treatment (A) The baseline T1-weighted image highlights significant sacroiliitis with evident joint inflammation, effusion, and structural abnormalities. These findings are consistent with active disease and inflammatory damage prior to treatment initiation. (B) The follow-up T1-weighted image taken after 12 months of infliximab therapy demonstrates a marked resolution of inflammation and significant improvement in joint integrity, with no new erosive changes observed. MRI: magnetic resonance imaging

**Figure 3:**
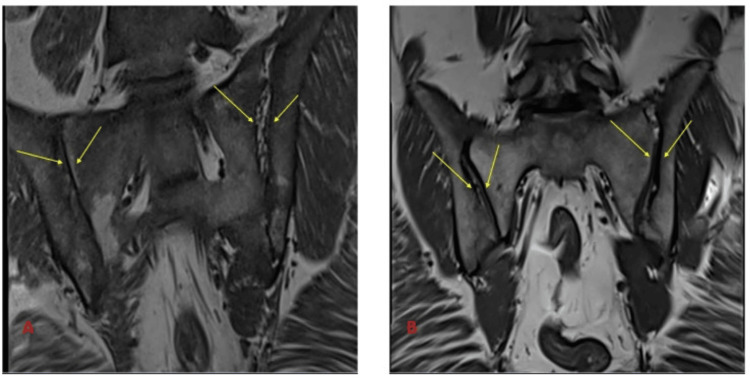
MRI T2-weighted image of sacroiliac joints before and after treatment (A) The baseline MRI T2-weighted image highlights significant sacroiliitis with evident joint inflammation, effusion, and structural abnormalities. These findings are consistent with active disease and inflammatory damage prior to treatment initiation. (B) The follow-up MRI T2-weighted image taken after 12 months of infliximab therapy demonstrates a marked resolution of inflammation and significant improvement in joint integrity, with no new erosive changes observed. MRI: magnetic resonance imaging

**Figure 4:**
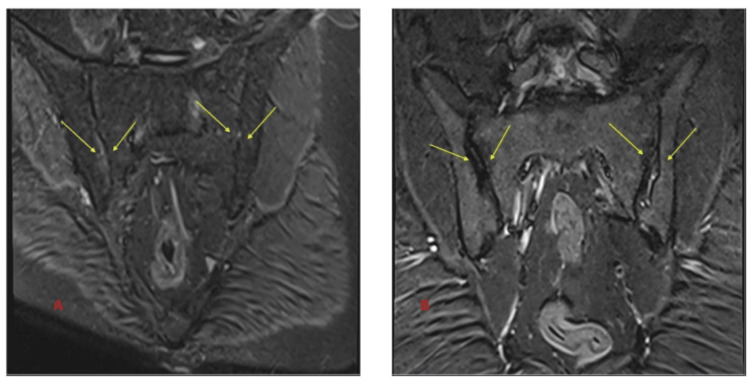
MRI STIR-weighted image of sacroiliac joints before and after treatment (A) The baseline STIR-weighted image highlights significant sacroiliitis with evident joint inflammation, effusion, and structural abnormalities. These findings are consistent with active disease and inflammatory damage prior to treatment initiation. (B) The follow-up STIR-weighted image taken after 12 months of infliximab therapy demonstrates a marked resolution of inflammation and significant improvement in joint integrity, with no new erosive changes observed. MRI: magnetic resonance imaging; STIR: short tau inversion recovery

MRI T1-weighted, MRI T2-weighted, and MRI short tau inversion recovery (STIR)-weighted images of sacroiliac joints before and after treatment are shown in Figure 2, Figure 3, and Figure 4.

## Discussion

The simultaneous occurrence of axial and peripheral joint involvement in a patient with psoriasis presents a significant diagnostic challenge, particularly in differentiating between axSpA and PsA [2]. Although classification criteria such as ASAS and CASPAR offer useful frameworks [9,10], they are not definitive diagnostic tests; rather, the overall clinical picture, including imaging, laboratory, and detailed joint assessments, must be considered [12].

Therapeutic efficacy of infliximab

Infliximab therapy in this patient led to marked improvements in both axial and peripheral disease activity [18]. Significant reductions in BASDAI, HAQ Disability Index, and VAS for pain, alongside improved grip strength, indicate a beneficial impact on both inflammation and peripheral joint function [18].

Alternative treatment considerations

While TNF inhibitors remain the first-line treatment for both axSpA and PsA [20], alternative biologic agents are available. Interleukin-17 (IL-17) inhibitors (such as secukinumab and ixekizumab) have demonstrated efficacy, particularly in patients with axial manifestations and TNF-resistant disease [21]. In some cases, IL-23 inhibitors (e.g., guselkumab and ustekinumab) may also be considered for patients with pronounced peripheral symptoms and severe skin involvement [22].

Role of imaging and limitations

Serial MRI was crucial in monitoring the resolution of sacroiliac inflammation and guiding treatment efficacy [14]. However, the absence of dedicated peripheral joint imaging (such as ultrasound or MRI for synovitis) represents a limitation; future studies should integrate comprehensive imaging assessments to better differentiate between axSpA with incidental psoriasis and true overlapping PsA [2].

Clinical significance and future directions

This case highlights the importance of a comprehensive, long-term treatment approach in managing complex spondyloarthropathies with overlapping features [3]. Future research involving larger cohorts and dedicated peripheral joint imaging is essential to refine diagnostic accuracy and therapeutic options [4].

## Conclusions

Our case report highlights the effectiveness of infliximab in improving both axial and peripheral symptoms in a patient with overlapping features of axSpA and PsA. The treatment resulted in significant reductions in BASDAI, HAQ, and VAS scores, along with improvements in grip strength. This report is notable for its comprehensive functional assessments and advanced imaging techniques, which provide a detailed evaluation of treatment response. The rigorous diagnostic approach using ASAS and CASPAR criteria clarifies the challenges in diagnosing patients with overlapping axSpA and PsA features. The findings support the potential for more personalized treatment strategies and underscore the need for further studies to refine diagnostic accuracy and compare therapeutic options in this complex patient group.
